# A Weighted Decision-Level Fusion Architecture for Ballistic Target Classification in Midcourse Phase

**DOI:** 10.3390/s22176649

**Published:** 2022-09-02

**Authors:** Nannan Wei, Limin Zhang, Xinggan Zhang

**Affiliations:** School of Electronic Science and Engineering, Nanjing University, Nanjing 210023, China

**Keywords:** ballistic missile defense, target classification, multi-sensor data fusion, online feature evaluation, weighted decision-level fusion

## Abstract

The recognition of warheads in the target cloud of the ballistic midcourse phase remains a challenging issue for missile defense systems. Considering factors such as the differing dimensions of the features between sensors and the different recognition credibility of each sensor, this paper proposes a weighted decision-level fusion architecture to take advantage of data from multiple radar sensors, and an online feature reliability evaluation method is also used to comprehensively generate sensor weight coefficients. The weighted decision-level fusion method can overcome the deficiency of a single sensor and enhance the recognition rate for warheads in the midcourse phase by considering the changes in the reliability of the sensor’s performance caused by the influence of the environment, location, and other factors during observation. Based on the simulation dataset, the experiment was carried out with multiple sensors and multiple bandwidths, and the results showed that the proposed model could work well with various classifiers involving traditional learning algorithms and ensemble learning algorithms.

## 1. Introduction

All ballistic missiles follow a common trajectory from launch to impact, which is divided into three phases: boost, midcourse, and terminal. Each phase has a different level of difficulty in implementing an intercept. It is hard to intercept in the boost phase as the interceptor is required to be inside the attack range within a few minutes while the missile engines are firing. The defensive technologies used in the terminal phase are usually the easiest to build because they require only short-range missiles and radars, but the main disadvantage of terminal attacks is that there may not be enough time to schedule all interceptions when countering many large-scale attacks. The midcourse phase represents the majority of the flight time of a ballistic missile, from minutes to the better part of an hour, depending on the range of the missile. The midcourse phase provides the best opportunity to intercept an incoming warhead and gives the defense system more time to observe and discriminate countermeasures from the targets.

The efficient identification of a true warhead is a prerequisite for accurate interception by defense systems. With the increasingly complex battlefield environment, the mature application of warhead attitude control technology, and the development of hypersonic weapons, traditional ballistic missile defense systems face great challenges. Modern high-tech warfare can be regarded as information warfare. When using multi-source information to describe all aspects of an incoming target, the defense system can identify the target more accurately, and more reliably [1,2].

Multi-sensor data fusion technology for target recognition can be represented at three different levels ([3], pp. 51–56): signal-level fusion, feature-level fusion, and decision-level fusion. 

Fusion at the signal-level applies a combination operator to each set of registered pixels, which correspond to associated measurements from each sensor. The merged signal has a higher quality than the original source. Then, feature extraction and pattern classification are performed to achieve target identification [4,5,6]. In feature-level fusion, the measurement signals of each sensor are first converted to the original source features and then merged as a new feature to classify the target. Classification of the merged feature is essentially a general pattern recognition problem [7,8]. Decision-level fusion involves performing signal preprocessing, feature extraction, and pattern classification locally and then establishing a preliminary conclusion about the observed target. The fusion center combines the recognition results from each sensor to obtain the final ballistic target identity description [1,9,10,11].

Generally, signal-level fusion requires a wide communication bandwidth if sensors are located on different platforms. Feature-level fusion can synthesize homogeneous or heterogeneous sensors, and the dimensionality of the merged feature is generally relatively high, which leads to difficulties in the subsequent pattern classification. The decision-level fusion structure has low communication bandwidth requirements and can asynchronously process echo signals, which makes it more appropriate for complex ballistic missile problems.

However, most of the research on multi-sensor fusion for ballistic target recognition ignores a critical problem; that is, the quality of the target echo signal is influenced by the environmental working methods, the physical factors, etc. The quality of echo signals of the same target received by the same radar sensor at different times may be quite different. For a new target, it is necessary to know how reliable the online feature provided by the sensor is and how credible the output of each sensor classifier is. Therefore, proper dynamic reliability and credibility evaluation methods are helpful in improving recognition performance. 

This paper considers the following two factors of the ballistic target classification problem in the midcourse phase: (1) each radar sensor has different working modes and signal resolutions, and (2) each sensor has different weights at each process time. Therefore, a weighted decision-level fusion architecture is proposed to take advantage of data from multiple radar sensors, for which an online feature reliability evaluation method is used to comprehensively generate sensor weight coefficients. 

The remainder of this paper is organized as follows. Section 2 introduces the background knowledge on the characteristics of ballistic missiles and radar observation for the recognition of a ballistic target. Section 3 and Section 4 provide a novel reliability evaluation algorithm and the proposed architecture. Section 5 illustrates the results of the experiment. Section 6 provides the discussion and summary. 

## 2. Radar Network System for Ballistic Target Classification

This section primarily describes the flight characteristics of ballistic targets and the characteristics’ wide-band and narrow-band radar observation signals. It then introduces the radar echo signal simulation method to prepare data for the subsequent multi-sensor fusion target classification research.

### 2.1. Ballistic Target Characteristics

**Figure 1:**
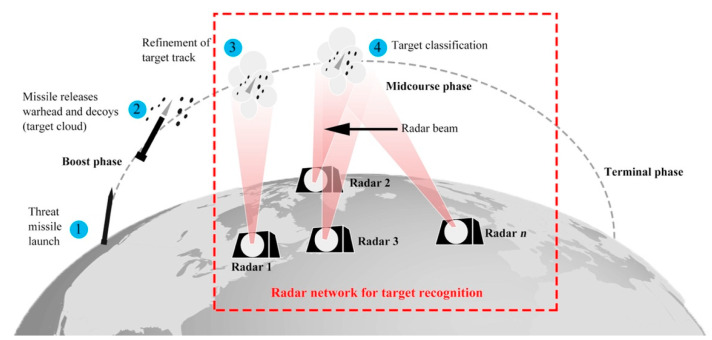
The scenario for a ballistic missile defense system includes a complex, global network of components. (1) The launch of the threat missile is detected by forward-based radars, (2) the threat missile releases its warhead and decoys, (3) the ground-based radar begins tracking the targets, (4) discrimination radars observe the target to try to determine which object is the warhead. The red dashed box highlights the specific functions that are addressed in this paper.

Figure 1 shows the general characteristics of ballistic missile flight, where the launch of the threat missile is detected by forward-based radars at (1), the threat missile releases its warhead and decoys at (2), the ground-based radar begins tracking the targets at (3), and discrimination radars observe the targets to try to determine which object is the warhead at (4).

Targets released by missiles in the midcourse phase follow the ballistic trajectory but have special micro-motion characteristics. Warheads and decoys move with precession motion due to the separation disturbance and retain this motion until they re-enter the atmosphere [12]. Compared to warheads and decoys, debris is lighter in weight and generally tumbles due to gravity and because of the absence of a spinning motor. 

**Figure 2:**
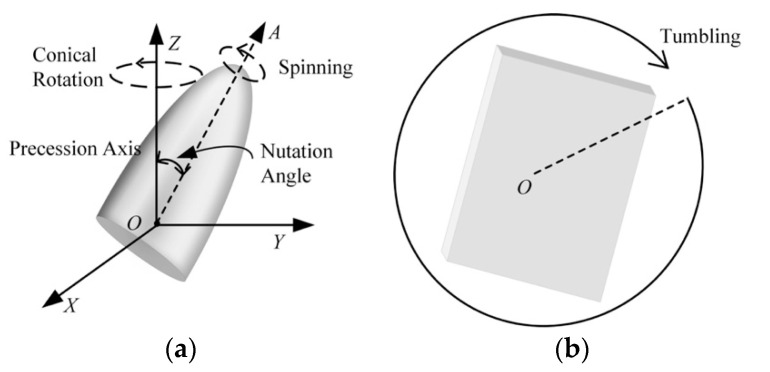
Micro-motion model of ballistic target: (**a**) precession motion; (**b**) tumbling motion.

Figure 2a illustrates a typical cone target with precession, where the precession motion can be viewed as a combination of two types of rotational motion: spinning of the target around its symmetry axis and conical rotation, such that the symmetry axis rotates conically around the precession axis. Cylinder debris tumbles around the precession axis illustrated in Figure 2b. The object micro-motion can be modelled by these parameters: spin frequency $\omega_{s}$, precession frequency $\omega_{p}$, tumbling frequency $\omega_{t}$, and nutation angle θ, which serve as a vital theoretical basis to distinguish different types of micro-motions. Chen [13] and Liu [14] give a more detailed mathematical analysis of the micro-motion of ballistic targets.

### 2.2. Radar Observation

Radar systems used for ballistic target recognition usually comprise low-resolution radars and high-resolution imaging radars. They have different advantages and complementary resources. Low-resolution radar can obtain radar cross section (RCS) time series, target polarization information, and micro-Doppler information [15]; these signals have good real-time recognition performance, but it is difficult to extract the fine features of the target. High resolution radar can achieve more accurate measurement of target structure and micro-motion information, but it is expensive and requires extensive processing resources. This study utilized RCS time series and high-resolution range profile (HRRP) time sequences.

#### 2.2.1. RCS Time Series

**Figure 3:**
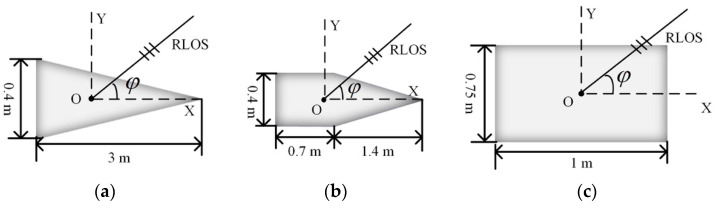
Sketch of three typical metal target models: (**a**) cone; (**b**) cone plus cylinder; (**c**) cylinder.

**Figure 4:**
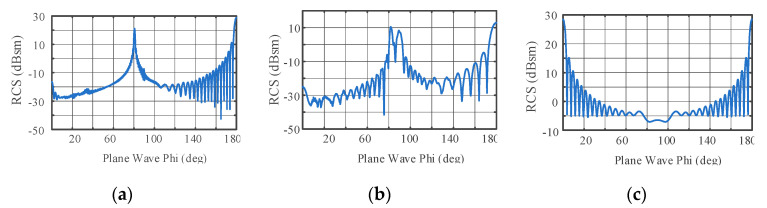
The full attitude-angle static RCS for *φ* ∈ [0°, 180° ]: (**a**) static RCS of cone; (**b**) static RCS of cone plus cylinder; (**c**) static RCS of cylinder.

RCS measurement is affected by factors such as scattering properties and the attitude of the target. For a given target, the value of the monostatic RCS is related to the incident wavelength and the observation angle, so the RCS can be defined as σ(f,φ,θ), where f is the frequency of the incident wave, φ is the elevation angle, and θ is the aspect angle [16]. Here, the aspect angle of the target is set as a constant value of $\theta_{0}$, so the RCS becomes $\sigma {\left(f,\varphi \right)}_{\theta_{0}}$. Three typical metal ballistic targets, shown in Figure 3, are considered here: cone, cone plus cylinder, and cylinder. Setting the frequency of the incident wave as 3 GHz and $\theta_{0}$ as $90^{{}^{\circ}}$, the scattering characteristics for different φ values for the three targets can be calculated with the Feldberechnung bei Körpern mit Beliebiger Oberfläch (FEKO), as shown in Figure 4. Considering $\varphi \in \left[50^{{}^{\circ}},80^{{}^{\circ}}\right]$, the usual range of observation of a defense radar, the RCS of the cylinder is the largest and the RCSs for the cone and the cone plus cylinder are similar, but the fluctuations in the RCS of the cone plus cylinder are more obvious than those of the cone.

#### 2.2.2. HRRP Sequences

HRRP is the sum of the projection vector of the sub-echo of the target scattering point along the radar line of sight (RLOS). When the bandwidth of the radar is so large that the distance resolution of the radar is much smaller than the size of the target, the equivalent scattering centers of the target are separated in the RLOS. It contains information on the structure, size, and shape of the target. It is important to effectively acquire and use this information in the field of ballistic target recognition. In addition, HRRP can also be used to extract the target radial length feature. 

**Figure 5:**
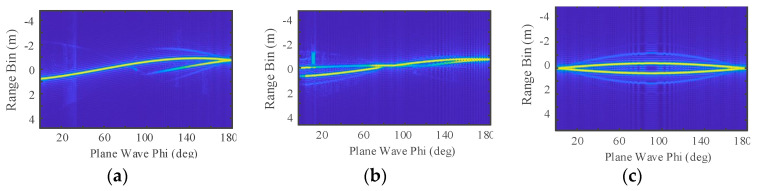
Normalized HRRPs of three models for $\varphi \in \left[0^{{}^{\circ}},180^{{}^{\circ}}\right]$: (**a**) HRRPs of cone; (**b**) HRRPs of cone plus cylinder; (**c**) HRRPs of cylinder.

HRRP can be obtained by inversely Fourier-transforming the frequency of the target, which can be written as:

(1) $$X\left(f\right)=\overset{K}{\underset{k=1}{\sum }}B_{k}e^{-j\frac{4\pi R_{k}}{c}f}$$

where f is the frequency, c is the speed of light, K is the number of scattering points on the target, and $B_{k}$ and $R_{k}$ are, respectively, the amplitude of the kth scattering point and the range between the kth scattering point and the observation radar at a certain time. Setting the radar center frequency $f_{0}$ as 9.5 GHz, the frequency step Δf as 15.625 MHz, and the range sample number N as 64, the number of visible scattering points varies with the radar line of sight for the three targets, as shown in Figure 5, where the cylinder has the highest number of visible scatter points and the cone has the smallest in $\varphi \in \left[50^{{}^{\circ}},80^{{}^{\circ}}\right]$.

### 2.3. Radar Echo Signal Generation

**Figure 6:**
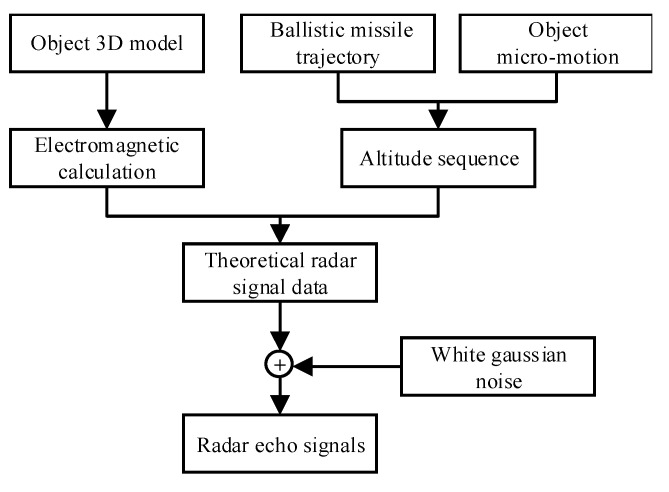
Flowchart for radar echo signal simulation.

The generation of radar echo signals by the scheme is depicted in Figure 6. It begins with three basic components: the object 3D model, the ballistic missile trajectory, and the object micro-motion. The details of the process are introduced below.

The typical types of ballistic targets in the midcourse phase are warheads, decoys, and debris. The 3D models shown in Figure 3 are used to represent these targets. The monostatic RCSs at the different carrier frequencies of the targets was computed with FEKO software.

**Figure 7:**
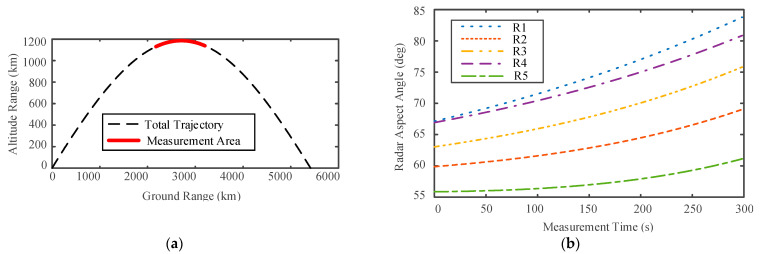
Ballistic missile trajectory and radar network simulation data: (**a**) model of ballistic missile trajectory; (**b**) multiple radar observation angle sequence.

The ballistic trajectory was formulated using the Systems Tool Kit (STK). The STK establishes the missile launch scene. The missile is launched from point A (44, 60) and lands at point B (110, 42). Five radars are located along the trajectory to observe the targets, and their positions are R1 (85,45), R2 (95,45), R3 (94,45), R4 (85,40), and R5 (95,40), respectively. The types of radar and the pulse repetition frequency (PRF) are shown in Table 1, including two narrowband radars and three wideband radars. Figure 7a illustrates the simulation ballistic missile trajectory, with radars measuring the ballistic target from 540 s to 820 s after its launch, as shown with the red bold line. The five radars’ observation angles as functions of the time sequence are shown in Figure 7b.

From practical experience, the nutation angle of the warhead is small due to attitude control, and the precession axis is often the reentry direction. In contrast, the nutation angle of the decoy is large. Debris usually presents a tumbling motion. Therefore, eight targets with various types and micro-motions were simulated and classified as four kinds according to the motion characteristics of the ballistic target, as shown in Table 2.

The target attitude time series can be obtained from the missile trajectory and target micro-motion using STK simulation. Then, theoretical echo signals can be obtained by combining the attitude time series and electromagnetic calculation results. Finally, complex white Gaussian noise with an SNR of 12 dB can be added to the theoretical radar echo signals to obtain the radar observation echo signals.

## 3. A Novel Online Feature Reliability Evaluation Based on Dependence

As is well known, the quality of the echo signal received by the radar changes in a real-world online environment, and these constraints and correlations that exist in the physical world can be seen as dependencies. Therefore, Bayes’ theorem can be used to establish a dependence measure, which can automatically adjust to various conditions and track the characteristics of a dynamic system for ballistic target classification.

For an input feature vector x of a newly arriving target, the online feature reliability is denoted as

(2) $$r^{n}\left(x\right)=p(c\left(x\right)=\theta_{p}|\hat{c}\left(x\right)=\theta_{p})$$

which expresses the conditional probability that x potentially belongs to the class $\theta_{p}$ when it is classified to the class $\theta_{p}$ by the radar’s classifier $M_{n}$. $c\left(x\right)=\theta_{p}$ denotes the true class of x, and $\hat{c}\left(x\right)$ represents the predicted class declared by $M_{n}$.

Training information is used to measure $r^{n}\left(x\right)$. The neighborhood patterns of x in the training feature space $\chi_{n}$ generally have close attribute values. The *k-*nearest neighbors of x ($x_{k}$, k=1,⋯,K) are found first in the training feature space $\chi_{n}$ according to the Euclidean distance. $p(c\left(x\right)=\theta_{p}|x_{k})$ is used to expresses the probability that sample $x_{k}$ comes from the class $\theta_{p}$ and is obtained through following dependence formula:

(3) $$p\left(\theta_{p}|\theta_{j}\right)=\overset{K}{\underset{k=1}{\sum }}p(c\left(x_{k}\right)=\theta_{p}|x_{k})p(x_{k}|\theta_{j})$$

where the conditional probability $p(x_{k}|\theta_{j})$ is the probability of the neighbor sample $x_{k}$ when drawn from the class $\theta_{j}$. When the item $x_{k}$ is regarded as a random variable, Equation (3) is written as a multiplication between a coefficient matrix A and a vector y:

(4) Ay=b

where A is a C×K matrix,

(5) $$A=\left[\begin{array}{ccc} p(x_{1}|\theta_{j}) & \cdots & p(x_{K}|\theta_{1})\\ \vdots & \ddots & \vdots \\ p(x_{1}|\theta_{C}) & \cdots & p(x_{K}|\theta_{C}) \end{array}\right]$$

and b is a C×1 column vector containing the right sides of the conditional probability:

(6) $$b={\left[\begin{array}{ccc} p\left(\theta_{p}|\theta_{1}\right) & \cdots & p\left(\theta_{p}|\theta_{C}\right) \end{array}\right]}^{T}$$

and

(7) $$y={\left[\begin{array}{ccc} p\left(\theta_{p}|x_{1}\right) & \cdots & p\left(\theta_{p}|x_{K}\right) \end{array}\right]}^{T}$$

Depending on *K* unknown variables and *K* equations, solutions to the normal system of linear equations can be found as follows:

(8) $$y=A^{-1}b$$

Finally, the online feature reliability $r^{n}\left(x\right)$ is obtained by calculating the mean of *K* dependence probabilities:

(9) $$r^{n}\left(x\right)=p\left(c\left(x\right)=\theta_{p}|x\right)=\frac{1}{K}\overset{K}{\underset{k=1}{\sum }}p(\theta_{p}|x_{k})$$

Supposing that the training data of each class fits a Gaussian distribution, the neighbor sample $x_{k}$ is assigned to a class $\theta_{j}$ only with its intensity level, and the conditional density function is defined by:

(10) $$p(x_{k}\;|\theta_{j})=\frac{1}{{\left(2\pi \right)}^{\frac{d}{2}}{\left|\epsilon_{j}\right|}^{\frac{1}{2}}}\times \exp\left(-\frac{1}{2}{\left(x_{k}-u_{j}\right)}^{T}\epsilon_{j}^{-1}\left(x_{k}-u_{j}\right)\right)$$

where $u_{j}$ and $\epsilon_{j}$ are, respectively, the mean *d-*vector and the d×d covariance matrix associated with $\theta_{j}$ (*d* is the dimension of the feature vector). The elements $p\left(\theta_{p}|\theta_{j}\right)$ of b represent a conditional probability obtained through the confusion matrix of the classifier $M_{n}$, which can be regarded as expert experience.

(11) $$p\left(\theta_{p}|\theta_{j}\right)=\frac{q_{pj}}{\sum_{i=1}^{C}q_{ij}}$$

where the element $q_{pj}$ represents the number of samples that classifier $M_{n}$ predicts for the class $\theta_{p}$ in relation to the class $\theta_{j}$. 

## 4. Proposed Multi-Sensor Fusion Architecture for Ballistic Target Classification

**Figure 8:**
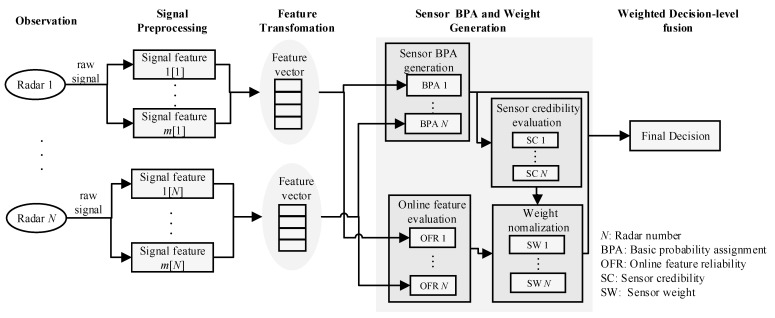
The workflow of our proposed multi-sensor fusion architecture for ballistic target classification.

The workflow of the proposed multi-sensor fusion architecture is described in Figure 8, and it comprises five stages as follows:

(a) Observation. This stage involves the collection of measured signals from the radar network, such as the RCS time series and the HRRP time sequence, in which the ballistic missile observation network is supposed to be comprised of N radars, i.e., $S=\left\{S_{1},\cdots ,S_{N}\right\}$.
(b) Signal preprocessing. At this stage, the radar echo signal is processed using feature extraction, and relatively stable and highly separable features are selected for identification. The intuition behind the signal preprocessing is to extract key signal features that depict the target details. In the proposed architecture, two measurement signals are mainly considered: the RCS time series and the HRRP time series. The RCS time series contains the following target characteristic information: (1) location characteristics, which describe the average location and specific location of the target RCS, such as the mean, quantile, minimum, and maximum; (2) dispersion characteristics, which indicate the dispersion of the target RCS sequence across the entire real number axis, such as the variance and standard deviation, standard mean deviation, and coefficient of variation; (3) distribution characteristics, such as the standard skewness coefficient and the standard kurtosis coefficient. HRRP sequences contain information about the structure, size, and shape of the target. It is important to effectively acquire and use this information in the field of ballistic target recognition. The use of HRRPs can make it possible to not only extract features, such as target distance and speed, but can also obtain target features, such as the number, position, and scattering intensity of the target. In addition, HRRPs can also be used to extract the target radial length feature. The features of the original signal extracted from the raw signal can be expressed as $A_{1},\cdots ,A_{d_{n}}$, where $d_{n}$ is the total number of the features of the sensor $S_{n}$.
(c) Feature transformation. At this stage, the signal features of each sensor are merged into a long vector. The dimension of the long feature vector is the sum of the data dimensions of the original signal features. Feature transformation reduces the impact of the high-dimensional feature space by removing redundant and irrelevant features. Principal component analysis (PCA), independent component analysis (ICA), and linear discriminant analysis (LDA) are popular methods for feature transformation. The transformation method is used for the radar signal feature vector of the radar $S_{n}$, and result is a new feature vector $F_{s}^{\prime }=\left[f_{1}\cdots f_{d_{n}^{\prime }}\right]$.
(d) BPA generation using trained classifier and sensor weight evaluation. At this stage, each piece of information extracted from the sensor is modeled as a basic probability assignment (BPA). The BPAs are generated based on the output of the trained classifier. Online feature quality evaluation and dynamic sensor credibility evaluation are used to obtain a comprehensive weight, which is used to modify the BPA of each sensor.
(e) Weighted decision-level fusion. At this stage, weighted decisions are made.

More details about stage (d) and stage (e) are introduced in the following section.

### 4.1. BPA Generation Using Trained Classifier

There are many mathematical theories available to represent the imperfection of data, such as Bayesian probability theory [17], fuzzy set theory [18], or belief function theory [19]. Most of these approaches can represent specific aspects of imperfect data. For example, probabilistic methods rely on probability distribution functions to express the uncertainty of the data. Fuzzy set theory introduces the novel notion of partial set membership, which enables imprecise reasoning. Belief function theory is a popular method for dealing with uncertainty and imprecision with a theoretically evidential reasoning framework. 

In our work, belief function theory is exploited to construct the evidence given by each radar because of its advantages in being able to separate the two sources of uncertainty and its fairly simple modeling of doubt and lack of information. Let $\Theta =\left\{\theta_{1},\cdots ,\theta_{C}\right\}$ represent the frame of discernment. The elements of the power set $2^{\Theta }=\left\{H|H\subseteq \Theta \right\}$ are called hypotheses. A basic probability assignment (BPA) defines a belief function m from $2^{\Theta }\to \left[0,\;1\right]$, satisfying:

(12) m(∅)=0

(13) $$\underset{H\subseteq \Theta }{\sum }m\left(H\right)=1$$

where ∅ denotes an empty set and H is any subset of Θ. The value taken by the BPA at H is called the basic probability mass and represents the accurate trust degree for the evidence for H in the recognition framework.

The sensor’s BPA is constructed from the likelihood of different classes of the output of $M_{n}$. Let $x=F_{s}^{\prime }$ be the input feature vector of the trained classifier $M_{n}$, and the output of the classifier is the posterior probabilities $\mu_{i}^{n}\left(x\right)$, i=1,⋯,C for each possible class. 

The $\mu_{i}^{n}\left(x\right)\in \left[0,\;1\right]$ represents the degree to which x belongs to the class $\theta_{i}$ according to the classifier $M_{n}$. Then, the likelihood value for each hypothesis under the framework Θ is defined by:

(14) $$l^{n}\left(\theta_{i}\right)=\mu_{i}^{n}\left(x\right)$$

and the likelihood of the unknown (the universal) set Ω is determined by:

(15) $$l^{n}\left(\Omega \right)=1-\max\left(l^{n}\left(\theta_{i}\right)\right)$$

We can normalize every likelihood to obtain the BPA:

(16) $$m^{n}\left(\theta_{i}\right)=\frac{l^{n}\left(\theta_{i}\right)}{l^{n}\left(\theta_{1}\right)+,\cdots ,+l^{n}\left(\theta_{C}\right)+l^{n}\left(\Omega \right)}$$

(17) $$m^{n}\left(\Omega \right)=\frac{l^{n}\left(\Omega \right)}{l^{n}\left(\theta_{1}\right)+,\cdots ,+l^{n}\left(\theta_{C}\right)+l^{n}\left(\Omega \right)}$$

where $m^{n}\left(\Omega \right)$ captures the total ignorant information about the classification undertaken by the classifier $M_{n}$ and plays a neutral role in the combination with the output of other classifiers.

### 4.2. Dynamic Sensor Weight Evaluation

Here, the online feature reliability and sensor credibility from the classifier performance are employed to determine the weights used in the given scenario. For an input feature vector x of a newly arriving target, each sensor obtains two reliability values: (1) $r^{n}\left(x\right)$, the reliability of the online feature; and (2) $\beta_{n}$, the credibility of the sensor.

The credibility of sensors is evaluated based on the degree of support between the basic probability assignments (BPAs) provided by sensors. We used the evaluation method proposed by Yong [20]. The sensor weight obtained after combining the two values is:

(18) $$v_{n}=r^{n}\left(x\right)\times \beta_{n}$$

and it is then normalized by:

(19) $$w_{n}=\frac{v_{n}}{\sum_{i=1}^{N}v_{i}}$$

### 4.3. Weighted Decision-Level Fusion

There are several decision-level fusion techniques, such as voting, weighted decision, Bayesian inference, the Dempster–Shafer method, generalized evidential processing theory, etc. The selection of an appropriate fusion strategy depends mainly on the output formats of the classifier. The purpose of this section is to obtain $m_{fused}$ using a variety of fusion techniques.

#### 4.3.1. Dempster–Shafer

The Dempster–Shafer method enables the fusion of several sources using the Dempster combination operator. Given two distinct BPAs in the set Θ, the aggregation can be achieved using the conjunctive combination rule [21]:

(20) $$m\left(H\right)=m_{1}\left(H\right)⊕m_{2}\left(H\right)=\frac{1}{K}\underset{A\cap B=H}{\sum }m_{1}\left(A\right)m_{2}\left(B\right)\;\;\;\forall A,B\subseteq \Theta$$

*K* is defined by:

(21) $$K=1-\underset{A\cap B=\emptyset }{\sum }m_{1}\left(A\right)m_{2}\left(B\right)\;$$

The normalization coefficient K evaluates the conflict between $m_{1}$ and $m_{2}$. The fused BPA $m_{fused}$ can be obtained by using Equation (20) to fuse the weighted BPAs of each sensor (N−1) times.

#### 4.3.2. Bayesian Inference

The Bayesian fusion structure uses a priori information on the probability that a hypothesis exists and the likelihood that a sensor can classify the data to the correct hypothesis [22]. The inputs to the structure are (1) $p\left(\theta_{j}\right)$, a prior probability that the object $\theta_{j}$ exists; (2) $p(D_{n,i}|\theta_{j})$, the likelihood that each sensor $S_{n}$ will classify the data as belonging to any one of the C hypotheses; and (3) $D_{n}$, the input decision from the *n*th sensor.

In accordance with the independence assumption, the estimated probability for the true class label $\theta_{j}$ can be calculated by

(22) $$p\left(D|\theta_{j}\right)=p\left(D_{1},\cdots D_{N}|\theta_{j}\right)=\overset{N}{\underset{n=1}{\prod }}p\left(D_{n}|\theta_{j}\right)$$

Denote by $p\left(D_{n}\right)$ the probability that the *n*th classifier labels *x* in the class $D_{n}\in \Theta$. *N* is the number of sensors and *C* is the number of classes, where $D=\left[D_{1}\cdots D_{N}\right]$ denotes the vector that generates the label of the ensemble. Then, the posterior probability needed to label **x** is

(23) $$p(\theta_{j}|D)=\frac{p\left(\theta_{j}\right)p\left(D|\theta_{j}\right)}{p\left(D\right)}$$

The denominator does not depend on $\theta_{j}$ and can be ignored, so the final support for class $\theta_{j}$ is

(24) $$m_{fused}\left(\theta_{j}\right)=p(\theta_{j}|D)\propto p\left(\theta_{j}\right)\overset{N}{\underset{n=1}{\prod }}p\left(D_{n}|\theta_{j}\right)$$

For each sensor’s classifier model, a C×C confusion matrix $CM^{n}$ is calculated from the testing dataset. $cm_{k,s}^{n}$ is the number of elements in the dataset whose true class label is $\theta_{k}$ and which is assigned by the classifier to class $\theta_{s}$. We denote $c_{k}$ as the total number of elements in the dataset from class $\theta_{k}$. Then,

(25) $$p\left(D_{n}|\theta_{k}\right)=\frac{cm_{k,s}^{n}}{c_{k}}$$

and prior knowledge $p\left(\theta_{k}\right)$ can be regarded as equal when unknown. Considering the sensor weight, the final support for class $\theta_{k}$ is

(26) $$m_{fused}\left(\theta_{j}\right)\propto \overset{N}{\underset{n=1}{\prod }}w_{n}\frac{\;cm_{k,s}^{n}}{c_{k}}$$

#### 4.3.3. Majority Vote

Voting is the simplest method and involves just counting the number of decisions for each class and assigning the object to the class that obtains the highest number of votes. The weighted voting fusion structure is described by:

(27) $$m_{fused}\left(\theta_{j}\right)=\overset{N}{\underset{n=1}{\sum }}\left[w_{n}m^{n}\left(\theta_{j}\right)+\frac{w_{n}m^{n}\left(\Omega \right)}{C}\right]$$

#### 4.3.4. Winner Takes All

The output of the most reliable sensor is taken as the judgment output:

(28) $$w_{k}=\underset{n=1,\cdots ,N}{\arg\;\max}w_{n}$$

(29) $$m_{fused}=m^{k}$$

### 4.4. Final Decision

A probability function must be constructed from the mass functions to make the final decision; that is, the one that maximizes the expected utility. The Pignistic transformation [23] is used and defined by

(30) $$BetP\left(H\right)=\underset{A\subseteq \Theta }{\sum }\frac{\left|H\cap A\right|}{\left|A\right|}\frac{m_{fused}\left(A\right)}{1-m_{fused}\left(\emptyset \right)}\;\;\;\forall H\subseteq \Theta$$

where |H∩A| is the cardinality of set |H|. Given $m_{fused}\left(\emptyset \right)=0$ and $\theta_{1},\cdots ,\theta_{C}\subseteq \Theta$, BetP(H) can be expressed as follows:

(31) $$BetP\left(\left\{\theta \right\}\right)=\underset{\theta \subseteq B}{\sum }\frac{m_{fused}\left(B\right)}{\left|B\right|}\;\;\;B\subseteq \Theta$$

and the unknown target for the class with the highest Pignistic probability can be obtained as follows:

(32) $$A^{*}=\underset{\theta \subseteq \Theta }{\arg\;\max}BetP\left(\left\{\theta \right\}\right)$$

## 5. Experimental Results

The proposed architecture was tested on a simulation dataset. Through the progressive simulation described in Section 2.3, a ballistic missile dataset with four types of ballistic targets measured by five radars was obtained. In this section, the experiment parameters and the results are described.

### 5.1. Experiment Setup

Firstly, the midcourse echo signal of the target was recorded over 280 s. Then, the records of each radar were sliced to form the signal samples, and the signal slicing window sizes are shown in Table 1 with a window step size of 1 s. Finally, the available datasets were randomly divided into training sets (four out of five of all the datasets) and a testing set (the remaining dataset). It is worth noting that each data splitting operation was synchronized for the five radars.

The signal features of each radar are shown in Table 3. In the feature transformation stage, PCA and ICA were exploited separately. In pattern classification, traditional learning algorithms and ensemble learning algorithms are applied to verify the adaptability of the proposed model. Traditional learning algorithms include decision tree (DT), *k*-nearest neighbor (*k*NN), and Gaussian naïve Bayes (NB) algorithms. Ensemble learning algorithms include bagging, random forest bagging (RFB), adaptive boosting for multiclass classification (AdaBoostM2), random subspace boosting (RSB), stacking, and linear programming boosting (LPB) algorithms. The classifiers’ output is a probabilistic score for each hypothesis class for an unknown object. In the experiment, the class scores from the stacking classifier are calculated using Equation (11), that is, the confusion matrix of the classifier is used to calculate the posterior probability of the target. The target scores of other classifiers are given by their respective classifiers. When the target score is obtained, the BPA of each sensor is calculated according to Equations (16) and (17). 

Due to the balanced sample size of each class in our simulation dataset, model performance was evaluated using the mean of the accuracy and the F1-score.

### 5.2. Accuracy of the Weighted Decision-Level Fusion Model

Table 4 shows the classification accuracy of the multi-sensor weighted decision-level fusion model, which uses the PCA method in the feature transformation stage. The result for a single sensor was obtained by directly inputting the transformed feature vector into the classifier. When comparing the five single sensors, R1 had the worst classification performance, while R2 had the best performance. Compared to R2, DS, BAYES, MV, and WTA, the average accuracy rates of the four fusion strategies increased by 4.43%, 8.46%, 8.23%, and 3.75%, respectively.

Table 5 shows the classification accuracy of the fusion model with the ICA method in the feature transformation stage. After weighted decision-level fusion, compared to R2, which had the best performance, the average accuracy rates of the four fusion strategies DS, BAYES, MV and WTA increased by 0.69%, 4.24%, 4.07%, and 0.32%, respectively.

### 5.3. F1-Scores of Classes

Table 6 shows the F1-scores of each class using the multi-sensor weighted decision-level fusion method with the PCA method in the feature transformation stage. Compared to R2, which had the best recognition performance, the F1-scores for the warheads increased by 3.31%, 9.19%, 8.77%, and 0.46%, respectively, with the four fusion strategies DS, BAYES, MV, and WTA.

Table 7 shows the fusion methods when using the ICA method in the feature transformation stage. Compared to R2, the warhead F1-score was improved by 0.14%, 5.29%, 4.77%, and −3.29%, respectively, with the four fusion methods DS, BAYES, MV, and WTA.

In summary, Bayesian fusion rules in the four types of fusion algorithms showed better performances; the reason may have been that the algorithm integrates the a priori probability of each class. The winner takes all method was not as stable as other fusion methods, and the reason may have been that it selects the output of the most reliable sensor each time, but the classification effect of the sensor affects the final judgment.

The most significant contribution of this work is that the accuracy of the ballistic target classification was increased by taking advantage of a combination of sensor data. Due to differences in working bandwidth, carrier frequency, and data rate, radar recognition performances can be quite distinct. As mentioned before, micro-motion feature extraction is an important means to distinguish the real warhead from other targets in the ballistic midcourse phase. It is easy to identify the debris, as it tumbles randomly and its motion form is single. The real warhead and the decoy have similar shapes and similar motion forms and, when only a single sensor observation is relied on, a certain degree of misjudgment about the true warhead can occur, especially when the data rate is relatively low. However, by using the comprehensive fusion model proposed in this study, it is obvious that the advantages of the radar systems can complement each other. The proposed model has good applicability, and it showed improved performances under different classification algorithms, where the average accuracy rate increased in the range from 0.32% to 8.46%, and the improvement in the F1-score for the warhead ranged from 0.14% up to 9.19%.

## 6. Discussion and Summary

Considering factors such as the different dimensions of the features between sensors and the different levels of recognition credibility of each sensor, a weighted decision-level fusion architecture using multiple radar sensors was proposed, and an online feature reliability evaluation method was also used to comprehensively generate sensor weight coefficients to overcome the deficiency of a single sensor and enhance the recognition rate for warheads in the midcourse phase.

Firstly, background knowledge on ballistic missiles was introduced. Then, the multi-sensor fusion architecture was described, which was divided into five stages: the observation stage, the signal preprocessing stage, the feature transformation stage, the sensor BPA and weight coefficient generation stage, and the weighted decision-level fusion stage. Finally, we described the experiment carried out under with multiple sensor locations and multiple bandwidths, which showed that the proposed model could work well with various classifiers, including traditional learning algorithms and ensemble learning algorithms. The average accuracy rate increased in the range from 0.32% to 8.46%, and the improvement in the F1-score of the warhead ranged from 0.14% up to 9.19%.

However, for ballistic target identification, the experiments and performances described above had limitations. Firstly, the model employs decision-level fusion at the highest level, and it is unnecessary to assume that five radars work simultaneously at each fusion node. If only one radar system is active in a fusion node, the online feature reliability calculation will use the sensor’s own training data and observations to optimize the BPA function, while the output of the fusion stage depends only on the BPA-adjusted sensor. Secondly, the fusion performance was only assessed through the limited ballistic simulation data, and it would be necessary to analyze the fusion performance of multi-track and multi-position data in the future. Finally, anti-missile operations presently have more requirements for the real-time performance of a system, with the micro-feature extraction and target imaging of ballistic targets often relying on long-term observations, but the evaluation metrics in this paper only considered the classification accuracy, so how to extract stable features and achieve maximum classification accuracy under the restrictions of the data rate, accumulation time, bandwidth, and other conditions is a problem that will be studied further.

## Figures and Tables

**Table 1:** Ground-based radar net parameters.

Radar	Work Type	Prf, Hz	Window Length, s
R1	Narrowband radar, carrier frequncy: 3 GHz	1	10
R2	Narrowband radar, carrier frequncy: 1.5 GHz	500	2
R3	Wideband radar, center frequency: 10.5 GHz, bandwidth: 1 GHz frequency interval: 15.625 MHz	1	10
R4	Wideband radar, center frequency: 10.5 GHz, bandwidth: 1 GHz frequency interval: 15.625 MHz	10	4
R5	Wideband radar, center frequency: 10.5 GHz, bandwidth: 1 GHz frequency interval: 15.625 MHz	500	2

**Table 2:** Target micro-motion parameters.

Class	3D Model Type	$\omega_{s},\;Hz$	$\omega_{p},\;Hz$	θ, deg
Warhead	Cone	3	1.5	5
	Cone plus cylinder	3	2	8
Heavy decoy	Cone	3	1.5	10
	Cone plus cylinder	3	1.5	12
Light decoy	Cone	3	2	15
	Cone plus cylinder	3	1.8	20
Debris	Cylinder	$\mathrm{Tumbling}:\;\omega_{t}=2\;\mathrm{Hz}$		90
	Cylinder	$\mathrm{Tumbling}:\;\omega_{t}=4\;\mathrm{Hz}$		90

**Table 3:** List of radar signal features.

Radar	Signal Type	Signal Feature	Total
R1	RCS time series	Mean, standard deviation, kurtosis, skewness, second-order central moment, third-order central moment, range, energy spectrum entropy, coefficient of variation, standard mean difference	10
R2	RCS time series	Mean, standard deviation, kurtosis, skewness, second-order central moment, third-order central moment, range, energy spectrum entropy, coefficient of variation, standard mean difference, period	11
R3	HRRP time series	Number of scattering points, skewness, target length, SVD principal component, entropy, echo power, irregularity, length change range, length change period	9
R4	HRRP time series	Number of scattering points, skewness, target length, SVD principal component, entropy, echo power, irregularity, length change range, length change period, precession frequency	10
R5	HRRP time series	Number of scattering points, skewness, target length, SVD principal component, entropy, echo power, irregularity, length change range, length change period, precession frequency	10

**Table 4:** Mean accuracy of independent sensor and fusion model with PCA (in %).

Classifier	Single Sensor					Proposed Fusion Model			
	R1	R2	R3	R4	R5	*M_DS_*	*M_BAYES_*	*M_MV_*	*M_WTA_*
DT	66.52	89.51	77.23	83.71	83.71	79.02	95.76	96.43	94.87
*k*NN	68.53	89.29	83.71	86.38	81.7	94.87	97.99	98.88	95.09
NB	64.06	69.87	64.51	62.72	69.42	80.8	91.52	88.62	75.45
Bagging	73.88	91.07	85.94	87.95	90.18	98.88	97.99	99.55	95.54
RFB	75.89	91.07	83.71	89.06	90.18	98.88	98.21	99.11	96.88
AdaBoostM2	59.38	75.67	49.55	63.39	72.32	84.82	94.42	89.06	79.69
LPB	66.96	99.55	87.05	93.97	97.54	99.78	100	100	98.66
RSB	66.96	95.76	69.87	72.1	92.63	98.21	95.98	98.21	94.42
Stacking	67.19	91.96	81.47	89.73	83.04	97.54	97.99	97.99	96.88
Average	67.71	88.19	75.89	81	84.52	92.53	96.65	96.43	91.94

**Table 5:** Mean accuracy of independent sensor and fusion model with ICA (in %).

Classifier	Single Sensor					Proposed Fusion Model			
	R1	R2	R3	R4	R5	*M_DS_*	*M_BAYES_*	*M_MV_*	*M_WTA_*
DT	67.19	92.19	82.81	84.15	89.73	78.35	97.1	98.44	97.54
*k*NN	66.29	98.66	89.06	89.96	91.52	96.88	99.78	99.55	98.44
NB	60.94	80.36	65.18	72.77	85.04	88.84	95.09	95.54	85.49
Bagging	73.44	96.88	85.94	91.07	92.41	99.78	98.66	99.55	97.32
RFB	75.45	99.11	89.51	91.96	90.85	100	98.88	99.78	98.21
AdaBoostM2	58.04	87.05	60.27	73.44	82.81	84.82	96.21	85.04	89.51
LPB	52.9	98.44	78.57	76.34	86.61	99.33	97.32	99.78	93.75
RSB	56.92	86.61	73.21	63.84	83.93	96.65	93.3	97.54	75
Stacking	66.74	98.21	85.04	89.51	89.51	99.11	99.33	98.88	99.33
Average	64.21	93.06	78.84	81.45	88.05	93.75	97.3	97.12	92.73

**Table 6:** F1-scores of four classes in the proposed model with PCA (in %).

Classifier	Class	Single Sensor					Proposed Fusion Model			
		R1	R2	R3	R4	R5	*M_DS_*	*M_BAYES_*	*M_MV_*	*M_WTA_*
DT	Warhead	47.75	86.32	75.21	68.49	72.17	69.59	92.64	93.33	92.04
	Heavy decoy	72.25	80.36	70.39	80.53	85.58	78.76	93.58	96.33	92.24
	Light decoy	64.6	92.45	65.7	85.46	77.53	75.79	96.86	96.07	95.15
	Debris	81.45	99.11	97.3	100	100	94.93	100	100	100
*k*NN	Warhead	44.21	87.61	84.43	74.11	69.16	90.76	96.43	97.78	92.73
	Heavy decoy	75.52	81.61	76.02	83.84	86.49	94.93	97.76	98.64	93.69
	Light decoy	67.3	90.83	75	88.18	71.19	94.01	97.78	99.11	95.15
	Debris	81.1	96.94	98.65	99.55	100	100	100	100	98.68
NB	Warhead	24.49	71.37	50.75	40.37	60.14	75.94	90.5	86.54	64.39
	Heavy decoy	71.08	53.54	54.04	51.95	73.96	79.7	86.96	86.17	69.27
	Light decoy	60.77	58.75	55.14	58.3	44.12	77.6	88.69	85.57	71.77
	Debris	79.72	92.95	97.78	100	100	87.84	100	95.73	94.12
Bagging	Warhead	49	89.18	87.76	75.7	82.19	97.78	97.76	99.11	92.66
	Heavy decoy	80.83	83.04	79.82	83.12	92.59	98.21	96.83	99.11	94.12
	Light decoy	71.7	93.02	77.57	92.51	86.08	99.55	97.37	100	95.69
	Debris	89.34	99.11	98.2	100	100	100	100	100	99.56
RFB	Warhead	57.71	89.47	84.17	77.93	82.51	98.21	97.35	98.2	95.02
	Heavy decoy	79.17	83.33	75	84.62	92.66	99.11	96.83	99.11	95.54
	Light decoy	74.77	92.52	76.99	93.33	85.71	98.21	98.67	99.11	97.35
	Debris	88.8	99.11	98.2	100	100	100	100	100	99.56
AdaBoostM2	Warhead	15.38	67.23	3.36	55.98	65.09	80.19	92.05	86.27	58.39
	Heavy decoy	63.93	50.59	48.12	24.64	90.74	78.23	90.65	82.35	78.07
	Light decoy	56.72	86.42	50.68	61.78	6.78	81.69	94.98	88.26	87.74
	Debris	78.85	90.32	96.04	100	100	99.55	100	100	88.19
LPB	Warhead	55.23	100	92.24	88.61	96.86	100	100	100	97.74
	Heavy decoy	62.98	99.11	76.6	87.74	96.04	99.55	100	100	97.78
	Light decoy	54.08	99.11	79.82	99.55	97.3	99.56	100	100	99.11
	Debris	94.69	100	100	100	100	100	100	100	100
RSB	Warhead	47.78	96.83	68.07	57	91.74	98.65	97.3	98.65	94.27
	Heavy decoy	67.46	91.7	61.73	66.08	91.89	96.4	92.17	96.4	92.58
	Light decoy	61.69	94.59	51.61	64.71	87.07	97.8	94.55	97.8	92.02
	Debris	83.65	100	95.2	100	100	100	100	100	98.68
Stacking	Warhead	44.55	90.21	81.39	79.64	71.86	96.86	96.89	97.32	95.15
	Heavy decoy	72.73	84.16	72.46	84.21	85.58	95.54	96.83	96.43	96.43
	Light decoy	70	93.52	74.26	95.07	75.22	97.78	98.23	98.21	95.93
	Debris	79.82	100	97.74	100	100	100	100	100	100

**Table 7:** F1-scores of four classes in the proposed model with ICA (in %).

Classifier	Class	Single Sensor					Proposed Fusion Model			
		R1	R2	R3	R4	R5	*M_DS_*	*M_BAYES_*	*M_MV_*	*M_WTA_*
DT	Warhead	49.06	88.45	84.75	71.36	86.22	68.06	95.81	97.78	96.89
	Heavy decoy	70.74	82.76	72.25	76.39	86.73	74	95.15	97.3	95.45
	Light decoy	67.62	97.25	75.6	88.5	85.97	78.76	97.39	98.67	97.8
	Debris	79.18	100	98.21	100	100	95.81	100	100	100
k NN	Warhead	43.69	98.64	92.77	79.25	87.18	94.12	99.56	99.56	98.67
	Heavy decoy	72.5	97.76	79.61	86.96	90.41	97.72	99.55	99.11	97.74
	Light decoy	67.69	98.68	83.12	93.04	88.58	95.81	100	99.55	97.78
	Debris	77.65	99.56	100	100	100	100	100	100	99.56
NB	Warhead	12.5	77.98	51.58	47.73	74.07	86.67	93.1	91.87	73.96
	Heavy decoy	71.16	57.71	53.66	64.44	91.15	89.45	92.31	94.42	83.76
	Light decoy	62.43	84.54	56.54	75.22	74.78	87.25	94.98	95.65	83.61
	Debris	71.15	100	98.25	100	100	91.43	100	100	99.11
Bagging	Warhead	53.81	95.65	87.8	82.41	86.88	100	97.78	99.55	95.65
	Heavy decoy	77.97	93.64	77.98	87.93	92.04	99.55	97.3	99.11	96.4
	Light decoy	69.91	98.2	80	93.75	90.67	99.56	99.56	99.56	97.27
	Debris	88.61	100	97.3	100	100	100	100	100	100
RFB	Warhead	53.47	98.18	89.54	83.64	82.95	100	97.82	99.55	96.52
	Heavy decoy	81.36	98.25	82.51	86.84	93.09	100	97.72	99.56	96.8
	Light decoy	75.68	100	86.12	97.32	87.39	100	100	100	99.55
	Debris	88.14	100	99.56	100	100	100	100	100	100
AdaBoostM2	Warhead	14.06	81.75	60.93	39.74	65.06	79.45	95.58	80.37	84.16
	Heavy decoy	63.79	71.84	21.52	63.29	90	73.39	92.31	73.39	84.08
	Light decoy	63.59	94.39	50.64	79.57	74.13	88.35	96.89	87.8	90.99
	Debris	67.71	100	96.46	100	100	99.55	100	100	98.68
LPB	Warhead	38.76	96.77	83.7	61.59	78.51	98.64	95.32	99.55	89.17
	Heavy decoy	49.5	96.97	59.62	60.1	84.91	98.68	94.39	99.56	88.12
	Light decoy	51.1	100	72.73	85.71	83.49	100	99.55	100	97.39
	Debris	75.6	100	97.72	100	100	100	100	100	100
RSB	Warhead	37.62	83	72.1	51.14	78.51	95.15	93.02	96	56.25
	Heavy decoy	62.34	73.43	59.26	51.16	86.49	95.5	88.31	96.83	72.2
	Light decoy	53.39	90.57	67.94	65.79	71.22	95.96	92.04	97.35	79.85
	Debris	71.07	99.11	91.6	85.47	98.68	100	100	100	83.58
Stacking	Warhead	46.85	99.1	87.22	78.7	83.76	98.65	99.11	98.21	98.67
	Heavy decoy	73.68	96.52	77.48	85.71	88.48	98.65	98.64	98.2	99.1
	Light decoy	69.03	97.27	77.88	93.1	85.97	99.56	99.56	99.11	99.56
	Debris	77.27	100	97.74	100	100	99.56	100	100	100

## References

[1] Maurer D.E., Schirmer R.W., Kalandros M.K., Peri J.S. (2006). Sensor Fusion Architectures for Ballistic Missile Defense. Johns Hopkins APL Tech. Dig..

[2] Wilson D.K., Pettit C.L., Lewis M.S., Mackay S., Seman P.M. (2008). Probabilistic Framework for Characterizing Uncertainty in the Performance of Networked Battlefield Sensors. Defense Transformation and Net-Centric Systems 2008.

[3] Liggins M., Hall D., Llinas J. (2017). Handbook of Multisensor Data Fusion: Theory and Practice.

[4] Lu K., Zhou R. (2016). Sensor Fusion of Gaussian Mixtures for Ballistic Target Tracking in the Re-Entry Phase. Sensors.

[5] Janczak D., Sankowski M. (2012). Data Fusion for Ballistic Targets Tracking Using Least Squares. AEU-Int. J. Electron. Commun..

[6] Cooperman R.L. Tactical Ballistic Missile Tracking Using the Interacting Multiple Model Algorithm. Proceedings of the Fifth International Conference on Information Fusion. FUSION 2002. (IEEE Cat. No. 02EX5997).

[7] Wu X., Zhou Y. Intelligent Processing Research for Target Fusion Recognition System Based on Multi-Agents. Proceedings of the 2010 International Conference on Computational Intelligence and Software Engineering.

[8] Choi I.-O., Kim S.-H., Jung J.-H., Kim K.-T., Park S.-H. (2019). Efficient Recognition Method for Ballistic Warheads by the Fusion of Feature Vectors Based on Flight Phase. J. Korean Inst. Electromagn. Eng. Sci..

[9] McCullough C., Dasarathy B., Lindberg P. Multi-Level Sensor Fusion for Improved Target Discrimination. Proceedings of the 35th IEEE Conference on Decision and Control.

[10] Dasarathy B., McCullough C. Intelligent Multi-Classifier Fusion for Decision Making in Ballistic Missile Defense Applications. Proceedings of the 37th IEEE Conference on Decision and Control (Cat. No. 98CH36171).

[11] Bhattacharyya A., Saraswat V., Manimaran P., Rao S. (2015). Evidence Theoretic Classification of Ballistic Missiles. Appl. Soft Comput..

[12] Bankman I.N., Rogala E.W., Pavek R.E. (2001). Laser Radar in Ballistic Missile Defense. Johns Hopkins APL Tech. Dig..

[13] Chen V.C., Li F., Ho S.-S., Wechsler H. (2006). Micro-Doppler Effect in Radar: Phenomenon, Model, and Simulation Study. IEEE Trans. Aerosp. Electron. Syst..

[14] Liu J., Li Y., Chen S., Lu H., Zhao B. (2017). Micro-Motion Dynamics Analysis of Ballistic Targets Based on Infrared Detection. J. Syst. Eng. Electron..

[15] Cun-qian F., Jing-qing L., Si-san H., Hao Z. (2015). Micro-Doppler Feature Extraction and Recognition Based on Netted Radar for Ballistic Targets. J. Radars.

[16] Wu B., Liu X. A Novel Approach for RCS Feature Extraction Using Imaging Processing. Proceedings of the 2006 CIE International Conference on Radar.

[17] Olshausen B.A. (2004). Bayesian Probability Theory.

[18] Zadeh L.A. (1996). Fuzzy Sets. Fuzzy Sets, Fuzzy Logic, and Fuzzy Systems: Selected Papers by Lotfi A Zadeh.

[19] Shafer G. (1976). A Mathematical Theory of Evidence.

[20] Yong D., Wenkang S., Zhenfu Z., Qi L. (2004). Combining Belief Functions Based on Distance of Evidence. Decis. Support Syst..

[21] Voorbraak F. (1989). A Computationally Efficient Approximation of Dempster-Shafer Theory. Int. J. Man-Mach. Stud..

[22] Kuncheva L.I., Bezdek J.C., Duin R.P. (2001). Decision Templates for Multiple Classifier Fusion: An Experimental Comparison. Pattern Recognit..

[23] Smets P. (2005). Decision Making in the TBM: The Necessity of the Pignistic Transformation. Int. J. Approx. Reason..

